# Comparing anti-platelet and anti-thrombin therapies in the ischaemia–reperfusion injured coronary microcirculation of healthy and diabetic mice

**DOI:** 10.1007/s00395-026-01168-7

**Published:** 2026-04-02

**Authors:** Joanne L. Mitchell, Juma El-Awaisi, Dean Kavanagh, Bernhard Nieswandt, Robert F. Storey, Neena Kalia

**Affiliations:** 1 Microcirculation Research Group, Department of Cardiovascular Sciences, School of Medical Sciences, College of Medicine and Health, University of Birmingham, Birmingham, B15 2TT UK; 2 Institute of Experimental Biomedicine, University Hospital Würzburg, Würzburg, Germany; 3 Division of Clinical Medicine, University of Sheffield, Sheffield, UK; 4 NIHR Sheffield Biomedical Research Centre, Sheffield Teaching Hospitals NHS Foundation Trust, Sheffield, UK

**Keywords:** P2Y_12_ receptor inhibitors, Coronary microcirculation, Ischaemia–reperfusion injury, Platelets, Neutrophils, Hyperglycaemia, Intravital microscopy

## Abstract

Patients with type 2 diabetes mellitus (T2DM) face increased risk of heart failure after myocardial infarction (MI), despite successful PCI and dual anti-platelet therapy (DAPT), due to coronary microvascular obstruction. This study examined whether DAPTs provide vasculoprotective benefits in injured coronary microvessels, including in the setting of chronic hyperglycaemia. Mice were fed a normal (ND) or high-fat diet (HFD) for 16 weeks and treated with vehicle, aspirin plus ticagrelor, clopidogrel, prasugrel or cangrelor, anti-GPIbα antibody, or dabigatran. Intravital imaging assessed platelet, neutrophil, and fibrin presence in the beating heart subjected to ischaemia–reperfusion injury (IRI). Laser speckle contrast imaging evaluated overall ventricular perfusion, and infarct size was determined histologically. IRI increased platelet and neutrophil accumulation in coronary capillaries and reduced perfusion. DAPTs, particularly using prasugrel, and anti-GPIbα reduced platelet numbers but increased neutrophil infiltration. Despite limited perfusion improvement, infarct size decreased. Fibrin deposition was also extensive and contributed to platelet recruitment, as shown using dabigatran. HFD-fed mice demonstrated markedly elevated thromboinflammatory cell accumulation. DAPT with prasugrel reduced platelet and neutrophil presence, but left a significant residual presence of both. Despite perfusion improvements, infarcts remained larger. Our data do not support a simple linear relationship between reduced platelet microthrombi, improved perfusion, and infarct limitation. Whilst early platelet inhibition confers cardioprotection independently of flow recovery in healthy mice, metabolic compromise uncouples microvascular flow from myocardial tissue survival. This may explain the diminished cardioprotective efficacy of DAPTs in patients with T2DM and supports exploring combination vasculoprotective therapies targeting multiple microvascular perturbations.

**Supplementary Information:**

The online version contains supplementary material available at 10.1007/s00395-026-01168-7.

## Introduction

Primary percutaneous coronary intervention (PCI) for treating myocardial infarction (MI) focuses on rapidly re-establishing perfusion in the ischaemic myocardium by opening up occluded epicardial arteries. However, the beneficial effects of PCI can be limited by thrombotic events taking place within the implanted stent, with platelet release of thromboxane A_2_ and adenosine diphosphate (ADP) playing a critical role in this process. ADP interacts with platelet P2Y_12_ receptors, triggering a signalling cascade that plays a prominent role in amplifying platelet activation, aggregation and clot formation [16]. The use of dual anti-platelet therapy (DAPT), consisting of aspirin and a P2Y_12_ receptor inhibitor, has proved to be highly effective at preventing stent thrombosis as well as decreasing the risk of recurrent MI or stroke [30, 55].

However, despite successful PCI and DAPT, many patients still incur extensive muscle damage and develop heart failure after an MI [11, 33]. This may partly be due to unresolved problems and inadequate perfusion in the coronary microcirculation due to no actual ‘re-flow’ post-PCI, plaque embolisation and/or injury caused through microvascular reperfusion [23, 26, 35, 52]. Indeed, restoration of normal epicardial blood vessel flow but sub-optimal myocardial perfusion can be observed in as many as 50% of PCI patients, leading to worse outcomes than in patients with full perfusion recovery [15, 40]. Moreover, microvascular perfusion is worse in patients with type 2 diabetes mellitus (T2DM). This poses a significant clinical problem since the prevalence of T2DM in individuals undergoing PCI can be as high as 40% in developed countries [39, 41]. Diabetes predisposes the coronary microcirculation to sub-optimal perfusion post-PCI due to increased endothelial dysfunction, which exists in a pro-thrombotic and inflammatory state, and hyper-reactive platelets exhibiting intensified adhesion, activation and aggregation [22, 51, 57].

Whilst the ability of contemporary P2Y_12_ inhibitors to prevent platelet aggregation in larger coronary arteries is well established, less is known about their effectiveness at the level of the coronary microcirculation. This is because current clinical imaging tools, such as x-ray angiography, cannot spatially resolve blood vessels less than 200 μm in diameter. Therefore, it is not possible to directly image coronary microvascular dysfunction let alone determine whether DAPT effectively restores microcirculatory flow post-PCI. Nevertheless, clinical trials such as CV-TIME, PROMICRO-2 and REDUCE-MVI have assessed microvessel functional status and found it to either be unaffected or varyingly improved by P2Y_12_ inhibitors [38, 48, 62, 63]. This has been quantitated by assessing the index of microcirculatory resistance (IMR), an indirect measurement collected from an epicardial artery. As such, these studies do not visualise the microvessels nor provide direct information on the precise action of DAPTs within them. Limited experimental studies have also tested P2Y_12_ inhibitors in rat or mouse MI models, but these studies have not always combined them with aspirin and have focussed only on measuring infarct size as a primary outcome and not investigated microcirculatory effects [4, 37, 68].

The primary objective of this experimental study was to determine whether clinically used DAPTs could modify the platelet presence within the coronary microcirculation in vivo following ischaemia–reperfusion injury (IRI), and whether any anti-platelet effects are maintained in the setting of chronic hyperglycaemia. Using intravital microscopy and laser speckle contrast imaging in the beating heart, we directly visualised the effects of four clinically relevant P2Y_12_ inhibitors, namely clopidogrel, ticagrelor, prasugrel and cangrelor, administered with aspirin. These agents, selected for their distinct pharmacokinetic properties and reported pleiotropic actions beyond platelet inhibition, were hypothesised to differentially modulate coronary microvascular injury [53, 58, 60]. Hence, in addition to platelet accumulation, we assessed potential off target vasculoprotective effects of P2Y_12_ inhibition, including modulation of neutrophil recruitment, endothelial adhesion molecule expression, and oxidative stress. We then tested whether the most effective DAPT regimen preserved coronary microvascular perfusion in the setting of a diet-induced chronic hyperglycaemia. Coronary microvascular obstruction and no-reflow often persist after MI despite effective P2Y_12_ inhibition, suggesting involvement of additional pathological mechanisms such as platelet adhesion events upstream of P2Y_12_-dependent aggregation, as well as coagulation-driven, fibrin-rich microthrombus formation. These complementary mechanisms were explored using a GPIbα inhibitor and the direct thrombin inhibitor dabigatran. These agents were used as mechanistic tools, not therapeutic alternatives to DAPT, to define the relative contributions of platelet GPIb-mediated adhesion and fibrin formation to microvascular dysfunction following IRI.

## Methods

### Animals and myocardial ischaemia–reperfusion injury (IRI)

Experiments were conducted on male C57BL/6 mice (2–4 months) obtained from Charles River. All procedures received local approval from the Animal Welfare and Ethical Review Body (AWERB) and were conducted in accordance with the Animals (Scientific Procedures) Act of 1986 (HO Project licence P552D4447; conforms to guidelines from the Directive 2010/63/EU of the European Parliament on the protection of animals used for scientific purposes). Mice were fed either a normal chow diet (ND) or a high-fat diet (HFD; 60% kcal from fat; D12492; Research Diets) for 16 weeks [18]. The latter closely mimics the progression of T2DM in humans, leading to the development of obesity, hyperglycaemia and increased insulin secretion and resistance [14]. Plasma glucose levels were measured in HFD-fed mice after tail tip vein puncture using a Contour XT glucometer (Bayer).

Anaesthesia was induced by an intraperitoneal administration of ketamine hydrochloride (100 mg/kg) and medetomidine hydrochloride (100 mg/kg), and appropriate depth was confirmed by checking the pedal reflex every 15 min. Mice were intubated and ventilated with medical oxygen via a MiniVent rodent ventilator (stroke volume: 220 μL, respiratory rate: 130 breaths/min; Harvard Apparatus). The carotid artery was cannulated to facilitate infusion of saline, fluorescent dyes, antibodies and some anti-platelet agents. Sham surgery or myocardial IRI was performed as previously described prior to imaging of the beating heart [19, 21, 32]. To access the heart, the mouse was placed in a right lateral decubitus position and an incision was made in the left thoracic region to uncover the pectoral muscles. Muscle and connective tissue were carefully cauterised to expose the left rib cage. A thoracotomy between the third and fourth left ribs was performed, and the pericardium was bluntly opened. Surgical retractors were used to hold back the ribs so that the heart could be easily accessed. The left anterior descending (LAD) coronary artery was then identified and temporarily suture ligated (Ethicon, UK) for 45 min by tying the suture around plastic tubing, followed by reperfusion for either 2 h for intravital imaging, LSCI, tissue and blood analysis or 4 h for infarct measurement. Mice were then killed by cervical dislocation for tissue and whole blood collection.

### Anti-platelet and anti-coagulant treatments

P2Y_12_ inhibitors such as clopidogrel and prasugrel are pro-drugs requiring hepatic metabolic conversion to their active component. Hence, they needed administering before induction of ischaemia to ensure adequate platelet inhibition during the critical period of reperfusion injury [42, 45]. Mice, therefore, received either vehicle control (0.5% carboxymethylcellulose in dH_2_O) or aspirin ± clopidogrel, ticagrelor or prasugrel at 24 h and at 1 h prior to anaesthesia by oral gavage at a volume of 10 ml/kg and dosed as follows: aspirin 0.5 mg/ml (dose 5 mg/kg); ticagrelor 10 mg/ml (dose 100 mg/kg); clopidogrel 0.5 mg/ml (dose 5 mg/kg); prasugrel 0.3 mg/ml (dose 3 mg/kg). Cangrelor, a fast-acting P2Y_12_ inhibitor, was administered intra-arterially via the carotid cannula (dose 0.5–6 μg/kg/min) using a syringe pump at 2 μl/minute to mice that had been orally treated with aspirin as above at 24 h and at 1 h prior to anaesthesia. The fab fragments of the p0p/B antibody, used to block the platelet GP1bα receptor, were administered intra-arterially 35 min post-ischaemia at a volume of 1 mg/ml (dose 100 μg/mouse) [5]. The anti-coagulant dabigatran, an inhibitor of thrombin-induced platelet activation as well as fibrin formation, was administered by oral gavage 1 h prior to anaesthesia at a volume of 0.5 mg/ml (dose 5 mg/kg).

### Intravital microscopy

Real-time intravital imaging of the anaesthetised mouse beating heart was performed as previously described [19, 21, 32]. A custom-designed 3D printed stabiliser ring (internal diameter 2.25 mm; external diameter 4 mm) was placed using gentle pressure on the surface of the left ventricle (LV) to reduce motion in a small region of the heart downstream of the ligation site. For fibrin imaging, a modified steel suction stabiliser was used that applied gentle negative pressure manually using a syringe to suction attach it to the LV [18]. To simultaneously image endogenous platelets, neutrophils and/or fibrin, 20 μl of either an APC- or PE-labelled anti-mouse CD41 antibody (BioLegend), a PE anti-mouse Ly6G antibody (BioLegend) and/or Alexafluor 647-labelled fibrinogen (Thermofisher Scientific) were injected intra-arterially. In some mice, fluorescein isothiocyanate conjugated to bovine serum albumin (FITC-BSA) was injected at 2 h post-reperfusion to qualitatively assess functional capillary density (FCD) in at least three separate areas of the LV.

Intravital imaging was performed using an Olympus BX61W microscope equipped with a Nipkow spinning disk confocal head (Yokogawa CSU) and an Evolve EMCCD camera (Photometrics). 2 min images were captured from a single pre-selected region every 15 min, during a continuous 2 h period of reperfusion, allowing the dynamics of trafficking cells to be assessed. To confirm that the events captured in the continuously imaged primary area were representative of the overall LV, additional regions were imaged at the end of the intravital experiment (referred to as ‘extra areas’). Images were captured, stored and analysed using Slidebook 6 software (Intelligent Imaging Innovations, USA). To aid analysis, captured videos were subjected to post-acquisition image repair using an in-house software (Tify) in which out-of-focus frames were removed [31]. Images were quantified by thresholding images in Fiji software (Image J), and the mean area coverage of platelets, neutrophils, and fibrin was calculated and presented as arbitrary units (AU).

### Laser speckle contrast imaging (LSCI)

Overall LV blood perfusion was measured in the beating heart using LSCI [19–21]. Briefly, a moorFLPI-2 LSCI device (Moor Instruments, UK) was positioned approximately 30 cm above the retractor exposed heart. A near-infrared laser diode, which emitted a low powered laser after passing through a diffuser, was used to illuminate the heart. Using the freehand selection feature within the LSCI software, a demarked area of interest was drawn on the LV, downstream of the LAD artery ligation site, for collection of 1 min flux (perfusion) data during pre-ischaemia, ischaemia and at every 15 min post-reperfusion for 2 h. 1000 frames were captured at each time point using the manufacturer supplied image software (mFLPI2Measure V2.0; mFLPIReview V5.0) at a frame rate of 25 Hz and using spatial processing (sliding window, time constant: 0.1 s). LSCI flux data were then exported into an in-house written analysis software package called Speckle Analysis* (*SpAn; https://github.com/kavanagh21/SpAN). SpAn software allowed systolic (peaks) and diastolic (troughs) bi-phasic events to be identified and collated from the LSCI flux measurements and exported into Excel [20]. For comparisons between different experimental groups, only the average flux values from diastole were used to represent overall microcirculatory perfusion within the LV.

### Whole blood platelet activation analysis by flow cytometry

The ability of DAPTs to reduce activation of agonist-stimulated circulating platelets was assessed ex vivo by measuring platelet surface P-selectin expression with flow cytometry. Whole blood was taken into 3.8% sodium citrate at a 1:10 ratio from the inferior vena cava of sham, vehicle- or DAPT-treated IRI anaesthetised mice and added at a 1:10 ratio to modified Tyrode’s-HEPES buffer (134 mM NaCl, 2.9 mM KCl, 0.34 mM Na_2_HPO_4_, 1 mM MgCl_2_, 20 mM HEPES, 5 mM glucose, pH 7.4). This contained an APC-labelled anti-mouse CD41 antibody (1:50, BioLegend) and a FITC anti-CD62P (P-selectin) antibody (1:50; Emfret). Platelets were then left unstimulated (resting) or activated for 20 min at room temperature in the dark using either a PAR4 receptor agonist [thrombin receptor agonist peptide (TRAP); Ala-Tyr-Pro-Gly-Lys-Phe-NH_2_ trifluoroacetate salt; 3 μM; Sigma], a GPVI receptor agonist [collagen-related peptide (CRP); 5 μg/ml; Cambridge, UK], an indirect thromboxane A_2_ agonist [arachidonic acid; 150 μg/ml; Merck] or a P2Y_12_ receptor agonist [ADP; 10–20 μg/ml; Sigma]. Following agonist activation, blood was fixed with 2% formyl saline (diluted 1:20) for 10 min and further diluted in PBS (1:2.5) before collecting 10,000 events per sample on a Cyan^ADP^ flow cytometer. Data were collected, with gating (using appropriately labelled isotype controls) and compensation applied, and analysed using summit v4.3 software. P-selectin mean fluorescence intensity (MFI) was measured on CD41 + cells.

### Immunostaining and coronary endothelial cell analysis by flow cytometry

Frozen LV tissue sections (10 μm) were fixed in acetone and then incubated at room temperature for 1 h with a PE-labelled anti-CD31 antibody (1:100; Biolegend) and an Alexafluor 647-labelled anti-vascular cell adhesion molecule-1 (VCAM-1) antibody (1:100; Biolegend), a FITC-labelled anti-DNA/RNA oxidative damage antibody (1:100; Abcam) and/or the superoxide indicator dihydroethidium (DHE; 5 μM; Thermofisher Scientific). Images were captured using an EVOS FL microscope (Thermofisher Scientific) and software analysed by quantifying MFI (Fiji, ImageJ). Flow cytometry also analysed VCAM-1 and P-selectin on coronary endothelial cells (ECs). Briefly, harvested hearts were manually minced with a scalpel, added to 0.1% collagenase and rotated in an incubator at 37 °C for 15 min. The supernatant was removed and the digestion process was repeated two times. The pellet was then resuspended in ACK and MACS buffer for 3 min to lyse red blood cells and stop enzymatic activity, respectively, and then centrifuged at 10,000 rpm at 4 °C for 10 min. The pellet was added to 20 mL media and run several times through a 70 μm strainer to obtain single-cell suspensions [19]. Cells were then incubated for 1 h with a brilliant violet 411-labelled anti-CD31 antibody (1:100; BioLegend; label ECs), APC-labelled anti-CD106 (VCAM-1) antibody (1:100; BioLegend) or a FITC-labelled anti-CD62P (P-selectin) antibody (1:100; Emfret) and appropriate IgG controls. Following antibody incubation, cells were centrifuged at 10,000 g for 10 min, resuspended in PBS containing 10% foetal bovine serum and fixed for 10 min in 2% formyl saline. Data were collected for 100,000 events using a Beckman Coulter Cyan^ADP^ and gating and compensation set using Summit software v4.3. Data were analysed using FlowJo X 10.0.7r2.

### Myocardial infarct size

The LAD coronary artery was re-ligated 4 h after reperfusion, and 0.5% Evans blue dye (Sigma) was injected via the carotid artery to delineate the area at risk (AAR). Mice were then culled, and harvested hearts were scalpel sliced into three sequential sections downstream of the sutured region of the LAD artery and stained with 2,3,5-triphenyl tetrazolium chloride (TTC; Sigma) for 20 min and then fixed. Images were acquired using a stereomicroscope and analysed (FiJi, ImageJ) to quantitate the infarct size (TTC^neg^ white regions) as a percentage of the AAR (TTC^pos^ red regions/Evans blue^neg^ regions).

### Statistical analysis

Statistical analysis was performed using GraphPad Prism 10.4.2 (GraphPad Software Inc. USA). Unpaired *t *tests were used to analyse differences between two groups, and a one-way analysis of variance (ANOVA), followed by Dunnett’s post hoc test were used to analyse differences in individual dependent outcomes (e.g. platelet presence, neutrophil infiltration, flux, etc.) between IRI-treated groups and both sham- or vehicle-treated IRI controls. For intravital experiments that followed a time course, the area under the curve (AUC) was also calculated and used for subsequent analysis as a summation of the entire period. All data are presented as mean ± SEM with *p* < 0.05 considered significant. No adjustment was made for multiple measures, and so the results are considered hypothesis-generating.

## Results

### DAPTs do not fully restore ventricular perfusion or FCD in ND-fed mice

**Fig. 1:**
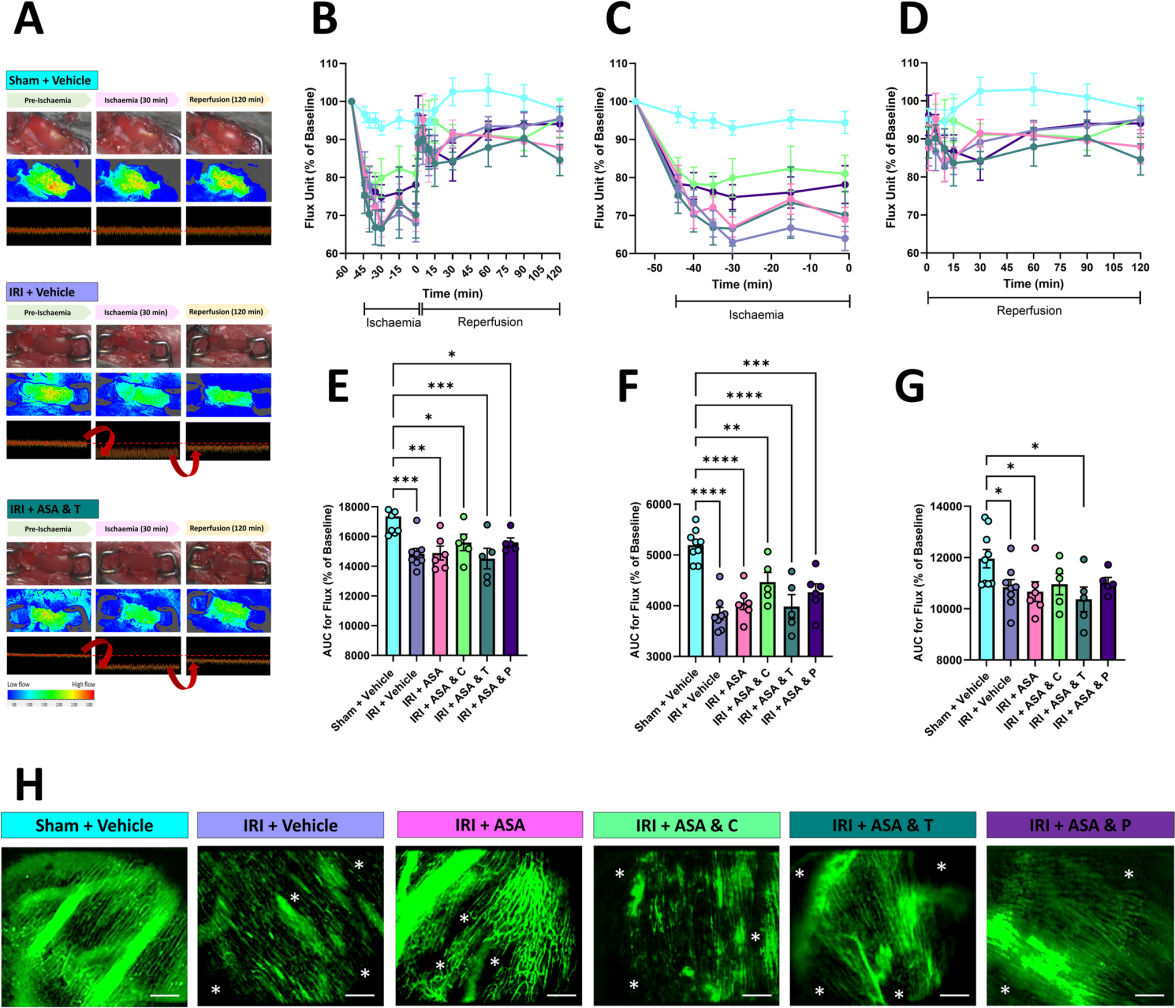
DAPTs do not fully restore ventricular perfusion or FCD following IRI. **A** Representative LSCI heatmaps and corresponding 1 min real-time flux readouts of the beating heart. Heatmaps show warm colours under basal conditions, cooler colours during ischaemia and warmer colours again as reperfusion is initiated—transition from warm to cool colours reflects a decrease in perfusion. Images for IRI + ASA and ticagrelor are shown (bottom), but similar images were obtained for all DAPT-treated experimental groups. **B**–**D** Quantitative time-course analysis of arbitrary flux unit readings as a percentage of baseline values obtained by LSCI during the entire IRI period (**B**), ischaemia period only (**C**) and reperfusion period only (**D**). **E**–**G** Corresponding area under the curves (AUC) for these graphs are shown below. *N* = 5–9/group. **H** Representative intravital images showing FITC-BSA perfused coronary microvessels at 120 min post-reperfusion. Patchy darker areas devoid of perfusion are indicated by asterisks (*). Scale bar represents 10 μm. N = 3/group. Graphs display mean ± SEM. *p < 0.05, **p < 0.01, ***p < 0.001, ****p < 0.0001 when tested using a one-way ANOVA followed by a Dunnett’s post hoc test

LSCI measured LV perfusion in a region downstream of the ligature site. As expected, heatmaps showed a rapid and striking transition to cooler colours in all mice, signifying greatly reduced perfusion as soon as artery ligation was performed, with concomitant and significant quantitative decreases in the arbitrary flux values. The heatmap colour and flux values failed to recover to pre-ischaemic baseline values in vehicle-treated IRI mice when the ligature was removed. Neither aspirin alone, nor in combination with any of the P2Y_12_ inhibitors, fully restored blood flow in injured hearts to baseline values during the reperfusion phase. However, blood flow recovered the most in mice receiving DAPT with clopidogrel or prasugrel (Fig. 1A–G).

Intravital imaging assessed coronary FCD after systemic FITC-BSA administration at 2 h reperfusion. An extensive network of FITC-BSA perfused capillaries was observed in sham mice, paralleling the arrangement of muscle fibres. IRI resulted in an increase in patchy areas devoid of FITC-BSA perfusion. Indeed, in some fields of view, diminished FCD was extensive as evidenced by no perfusion across much of the imaged area. Medium-sized blood vessels, if captured in the imaged area, remained perfused suggesting that blood flow in the smaller coronary capillaries was primarily affected by IRI. Neither aspirin alone nor in combination with any of the P2Y_12_ inhibitors improved FCD, with multiple patchy areas lacking capillary perfusion still discernible. FCD was most improved in mice receiving DAPT with prasugrel, with less areas devoid of perfusion observed (Fig. 1H).

DAPTs reduce, but do not fully abolish microthrombus presence and concomitantly increase neutrophil infiltration in ND-fed mice.

**Fig. 2:**
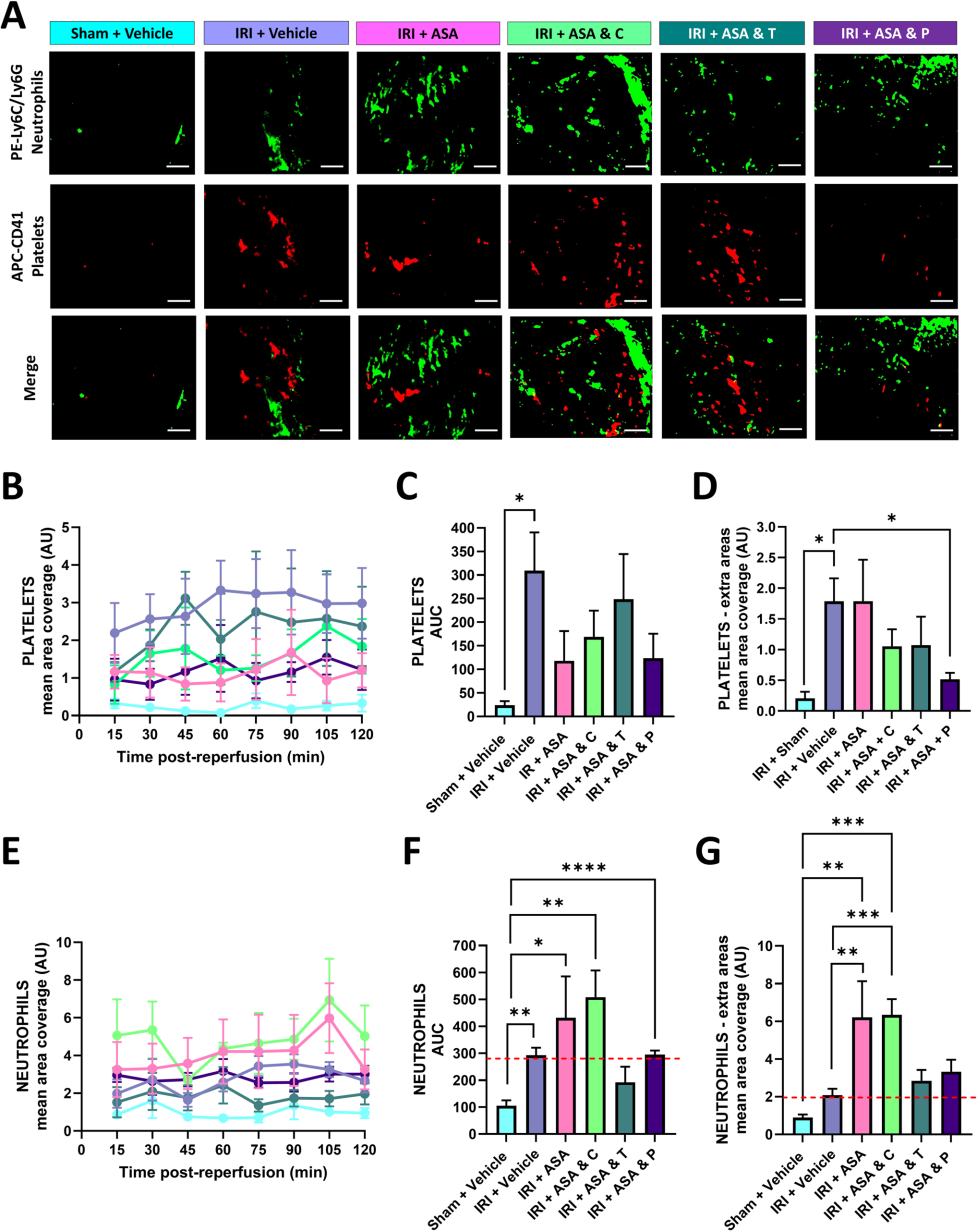
DAPTs reduce but do not fully abolish microthrombus formation and increase neutrophil infiltration following IRI. **A** Representative intravital images showing neutrophils (green) and platelets (red) at 120 min post-reperfusion. Scale bar represents 10 μm. **B**, **E** Quantitative analysis of intravital data for adherent platelets and neutrophils imaged continuously in a single region over a duration of 120 min post-reperfusion. **C**, **F** Corresponding area under the curves (AUC) for adherent platelets and neutrophils for this continuously imaged area. **D**, **G** Quantitative analysis of intravital data for adherent platelets and neutrophils imaged in extra areas at the end of experimentation. DAPTs generally increased neutrophil presence above that seen with IRI alone (red dashed line). *N* = 4–7/group. Graphs display mean ± SEM. *p < 0.05, **p < 0.01, ***p < 0.001, ****p < 0.0001 when tested using a one-way ANOVA followed by a Dunnett’s post hoc test

Intravital imaging demonstrated significant and rapid accumulation of platelet aggregates of varying size in injured coronary capillaries. Thereafter, the level of adherent microthrombi remained relatively constant over the imaged 2 h reperfusion period. Whilst DAPTs decreased platelet recruitment, in the continuously observed primary area and/or in the extra areas imaged at the end of the intravital experiment, they did not fully abolish their presence to levels observed in sham mice. DAPT with prasugrel was most effective (Fig. 2A–D).

Neutrophil adhesion, again occurring primarily within capillaries, also significantly and rapidly increased in response to injury. None of the DAPTs decreased neutrophil presence to levels seen in sham mice but, surprisingly, demonstrated a trend to increase their presence above that seen with IRI alone. Indeed, treatment with both aspirin alone and in combination with clopidogrel resulted in significant increases in neutrophil presence compared to injured mice receiving no therapy (Fig. 2A, E–G).

### DAPTs reduce agonist-induced platelet activation

**Fig. 3:**
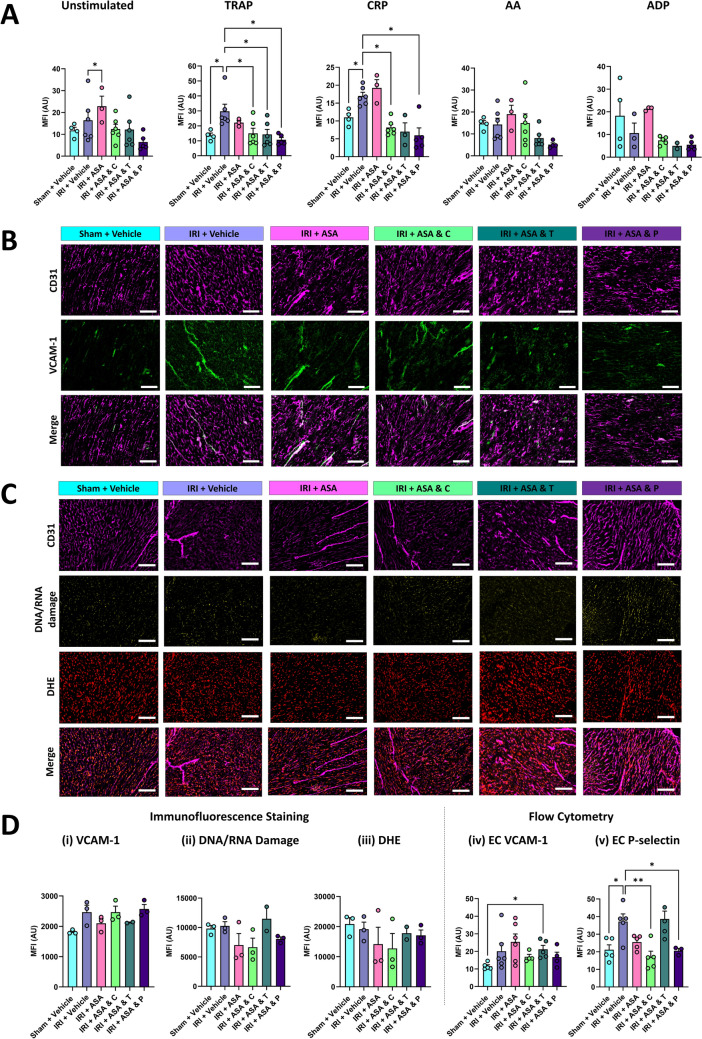
DAPTs reduce agonist-induced activation of circulating platelets. **A** Quantitative analysis of flow cytometry data for P-selectin exposure on the surface of resting (unstimulated) and agonist-stimulated platelets. P-selectin mean fluorescence intensity (MFI; arbitrary units (AU)) was measured on CD41^+^ platelets. **B** Representative immunofluorescence images of LV sections stained for CD31 (magenta) and VCAM-1 (green). Scale bar represents 100 μm. **C** Representative immunofluorescence images of LV sections stained for CD31 (magenta), DNA/RNA oxidative damage (green) and DHE (red). Scale bar represents 100 μm. Quantitative analysis of these immunofluorescence images is shown in (**Di**–**iii**). **Div**–**v** Quantitative analysis of flow cytometry data for VCAM-1 and P-selectin specifically on ECs obtained from digested hearts. *N* = 2–6/group. Graph displays mean ± SEM. *p < 0.05, **p < 0.01 when tested using a one-way ANOVA followed by a Dunnett’s post hoc test

Since DAPTs did not fully abolish microthrombus presence in injured coronary microvessels, their ability to effectively reduce the activation of circulating platelets at the doses used was assessed ex vivo. Flow cytometry was used to measure P-selectin exposure on the surface of unstimulated (resting) and agonist-stimulated platelets. The extent of platelet activation in resting platelets was not significantly greater in injured mice when compared to sham mice, suggesting platelets did not circulate in an activated state following IRI. In contrast, some agonist-stimulated platelets from injured mice demonstrated a greater activation status than platelets from sham mice. Notably, significant increases in response to TRAP and CRP were observed suggesting platelets may have been ‘primed’ during injury and subsequently circulated in a more readily activatable state if they encountered stimulating factors. Importantly, all DAPTs reduced the ability to activate platelets obtained from injured mice in response to agonist stimulation, confirming that they were functional in this model (Fig. 3A).

Myocardial inflammation was assessed by investigating VCAM-1 expression by both immunostaining of tissue sections and flow cytometrically on ECs isolated from digested hearts. There was a general trend for VCAM-1 to increase with IRI with no DAPT modifying its expression (Fig. 3B, D). Oxidative stress was also assessed by immunostaining for oxidative DNA/RNA damage and DHE expression, but neither significantly changed across the various groups (Fig. 3C, D). P-selectin expression was investigated flow cytometrically on digested heart ECs and was seen to significantly increase in response to IRI, again indicating a pro-inflammatory vascular status, with DAPT with clopidogrel and prasugrel most effective at reducing its expression (Fig. 3D).

### Targeting platelet GPIbα receptor is no better an alternative to DAPT with prasugrel

**Fig. 4:**
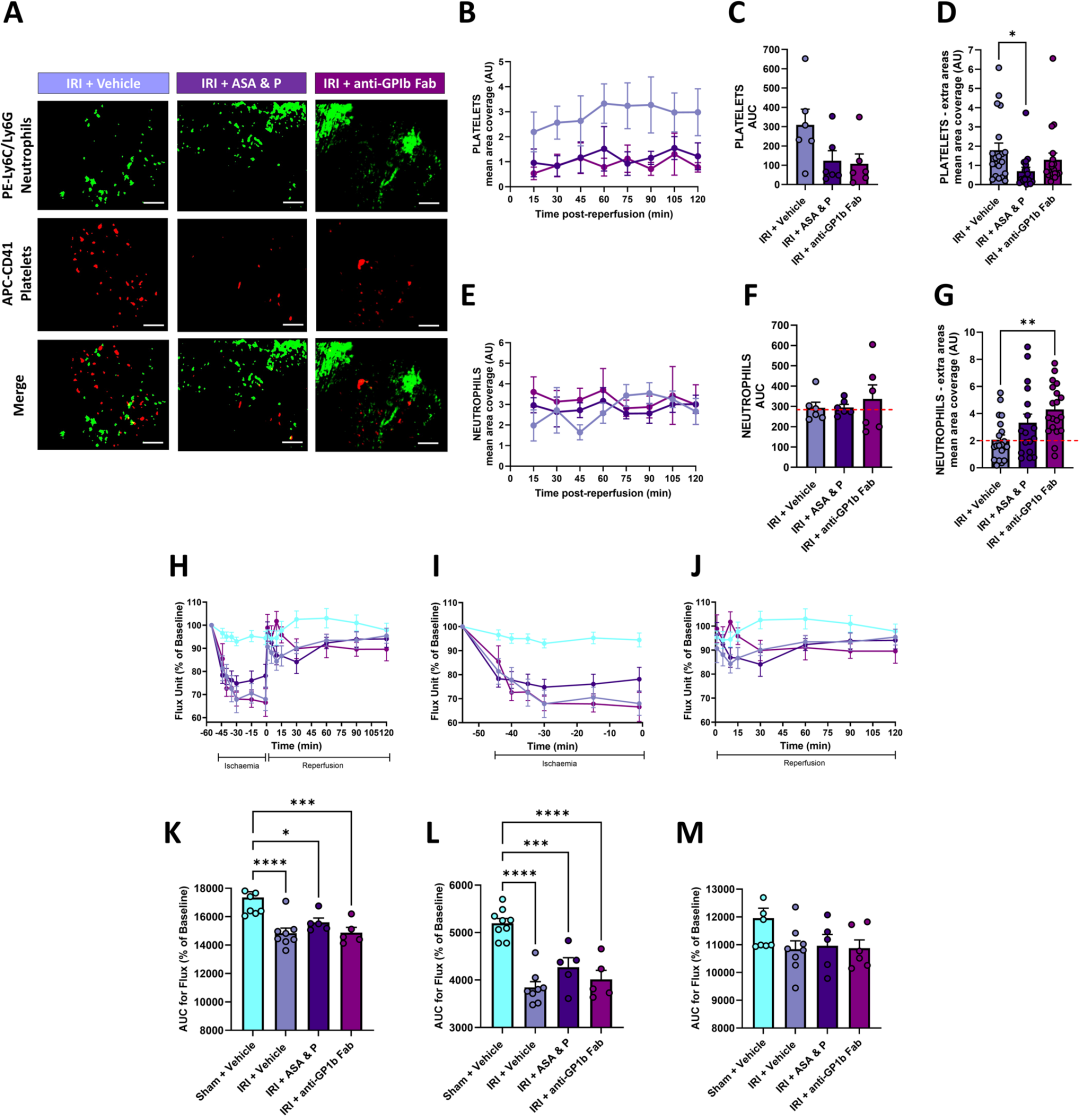
Targeting platelet GPIbα is no better an alternative to DAPT with prasugrel. **A** Representative intravital images showing neutrophils (green) and platelets (red) at 120 min post-reperfusion. Scale bar represents 10 μm. **B**, **E** Quantitative analysis of intravital data for adherent platelets and neutrophils imaged continuously in a single region over a duration of 120 min post-reperfusion. **C**, **F** Corresponding area under the curves (AUC) for adherent platelets and neutrophils for this continuously imaged area. **D**, **G** Quantitative analysis of intravital data for adherent platelets and neutrophils imaged in extra areas at the end of experimentation. Both strategies generally increased neutrophil presence above that seen with IRI alone (red dashed line). N = 6/group. **H**–**J** Quantitative time-course analysis of arbitrary flux unit readings as a percentage of baseline values obtained by LSCI during the entire IRI period (**H**), ischaemia period only (**I**) and reperfusion period only (**J**). **K**–**L** Corresponding AUCs for these graphs are shown below. N = 5–9/group. Graphs display mean ± SEM. *p < 0.05, **p < 0.01, ***p < 0.001, ****p < 0.0001 when tested using a one-way ANOVA followed by a Dunnett’s post hoc test

To determine whether targeting platelet activity via an alternative receptor to P2Y_12_ was more effective than DAPT with prasugrel, an anti-GPIbα Fab was also tested. Intravital imaging demonstrated that this strategy also decreased platelet presence, but again did not fully abolish their presence. In fact, in the extra areas imaged at the end of the experiment, targeting GPIbα was not as effective as DAPT with prasugrel (Fig. 4A–D). The anti-GPIbα Fab also increased neutrophil presence, with greater increases observed than with DAPT with prasugrel (Fig. 4A, E–G). LSCI showed that targeting GPIbα also did not fully restore overall ventricular perfusion in injured hearts to baseline values during the reperfusion phase (Fig. 4H–M). Since this strategy was no better than DAPT with prasugrel, we did not use it further in this study.

### Dabigatran reduces fibrin deposition which contributes to microthrombus formation

**Fig. 5:**
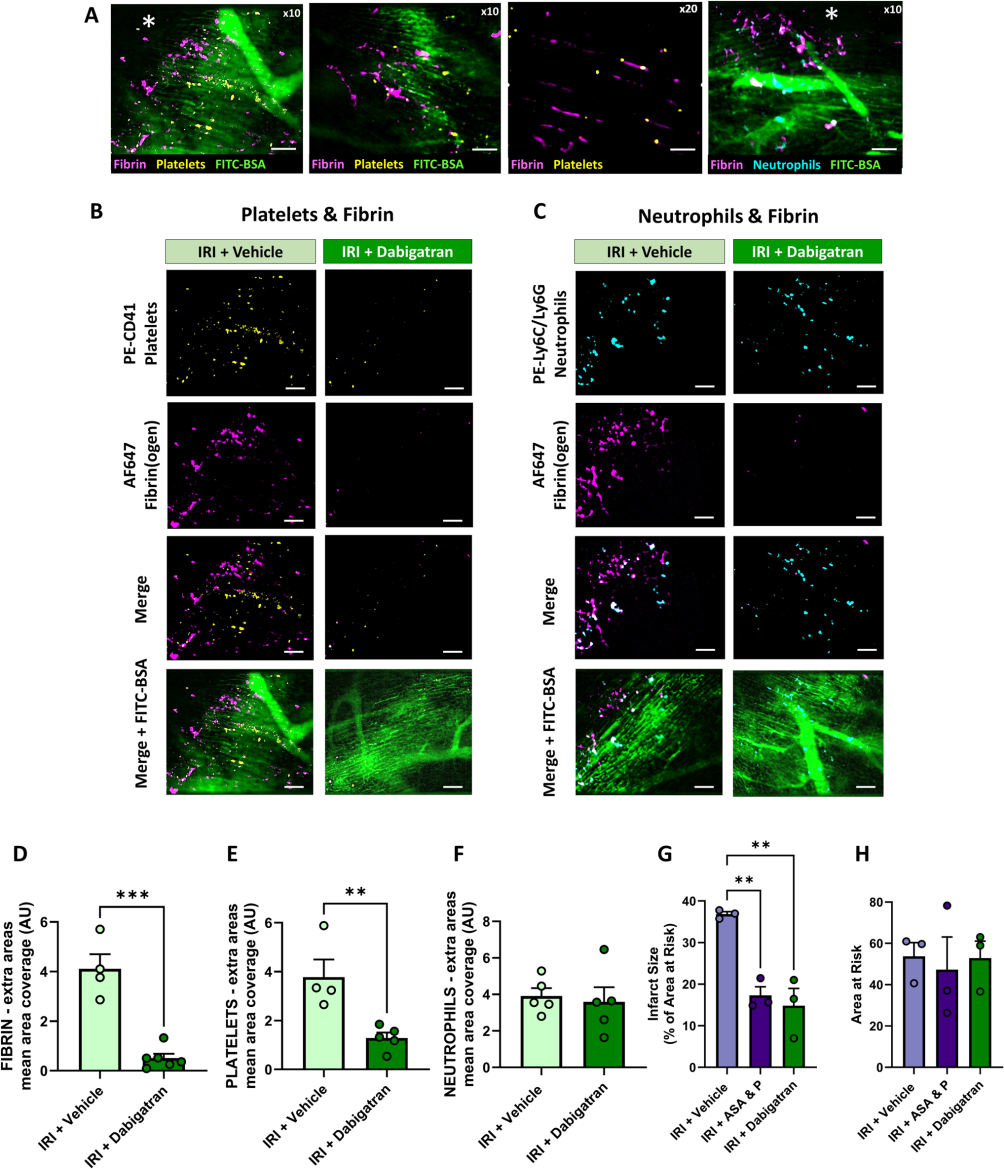
Dabigatran reduces fibrin deposition which contributes to microthrombus formation following IRI. **A** Representative intravital images from IRI mice showing fibrin (magenta), platelets (yellow), FITC-BSA (green) or neutrophils (cyan) at 15 min post-reperfusion. Fibrin appeared as either small, rounded clots or as elongated strands of protein that lined the length of multiple capillaries. Fibrin deposition was occlusive in some areas as evidenced by fibrin-rich areas upstream of patchy darker areas devoid of FITC-BSA as indicated by the asterisk (*). Scale bar represents 10 μm (× 10) and 5 μm (× 20). **B**, **C** Representative intravital images taken at 15 min post-reperfusion from mice treated with vehicle or dabigatran. Scale bar represents 10 μm. **D**–**F** Quantitative analysis of intravital data for fibrin, adherent platelets and adherent neutrophils imaged in extra areas at the end of experimentation. *N* = 4–5/group. **G**, **H** Quantitative analysis of infarct size and area at risk (AAR) determined by dual Evan’s blue and TTC-staining of heart sections. *N* = 3/group. Graphs display mean ± SEM. **p < 0.01, ***p < 0.001 when tested using an unpaired *t *test or a one-way ANOVA followed by a Dunnett’s post hoc test

Microthrombus formation within coronary microvessels could have been driven by fibrin deposition. Therefore, intravital imaging was used for the first time to detect fibrin presence in the IRI coronary microcirculation in vivo. Mice received fluorescently labelled fibrinogen which could be detected in the vasculature when converted into fibrin by thrombin. Fibrin appeared as small, rounded clots or as elongated strands of protein that lined the length of multiple capillaries. It only partially co-localised with platelets and neutrophils and commonly appeared on its own. Fibrin deposition was occlusive in some areas as evidenced by fibrin-rich areas upstream of darker areas devoid of FITC-BSA perfusion (Fig. 5A). To ascertain whether targeting fibrin formation could reduce platelet recruitment, some mice were pre-treated with dabigatran which significantly reduced fibrin deposition and platelet recruitment, with no effect on neutrophil accumulation (Fig. 5B–F).

### DAPT with prasugrel reduces infarct size

The ability of DAPT with prasugrel and dabigatran to modify myocardial infarct size was assessed. Both significantly reduced the infarct size when compared with IRI vehicle-treated mice. DAPT with prasugrel reduced the infarct size by 2.13-fold and dabigatran by 2.48-fold, values that were not significantly different from each other (Fig. 5G, H). DAPT with prasugrel achieved the greatest reduction in infarct size compared to all other DAPTs, although there was no significant difference between groups (aspirin alone = 2.03-fold, DAPT with ticagrelor = 1.53-fold, DAPT with clopidogrel = 1.79-fold—data not presented).

### DAPT with prasugrel is effective in HFD-fed mice, but does not modify infarct size

**Fig. 6:**
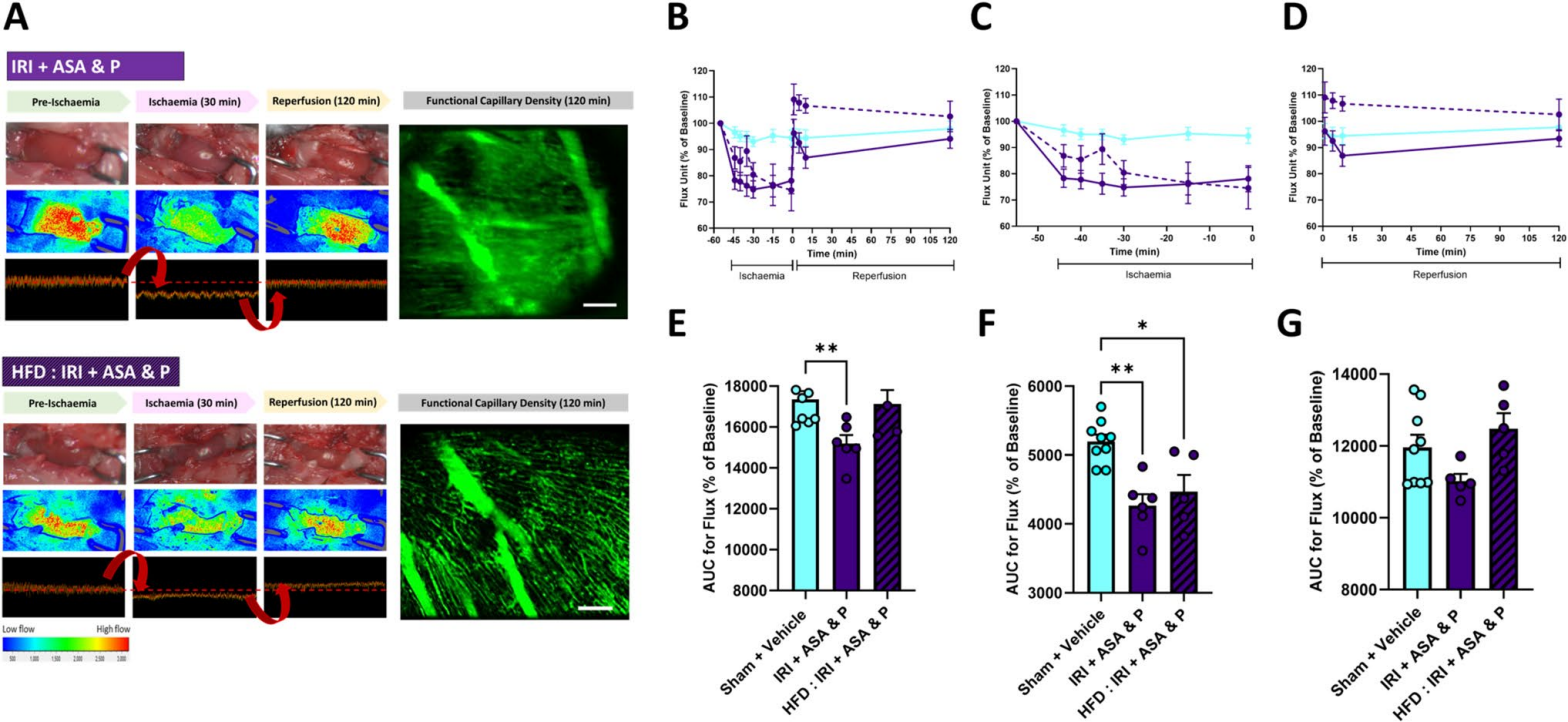
DAPT with prasugrel restores ventricular perfusion and FCD in the LV following IRI in HFD-fed mice. **A** Representative LSCI heatmaps and corresponding 1 min real-time flux readouts of the beating heart. Heatmaps show warm colours under basal conditions, cooler colours during ischaemia and warmer colours again as reperfusion is initiated—transition from warm to cool colours reflects a decrease in perfusion. Representative intravital images showing FITC-BSA perfused coronary microvessels at 120 min post-reperfusion (right). Scale bar represents 10 μm. (*N* = 3–6/group). **B**–**D** Quantitative time-course analysis of arbitrary flux unit readings as a percentage of baseline values obtained by LSCI during the entire IRI period (**B**), ischaemia period only (**C**) and reperfusion period only (**D**). **E**–**G** Corresponding area under the curves (AUC) for these graphs are shown below (*N* = 3–7). Graphs display mean ± SEM. *p < 0.05, **p < 0.01 when tested using a one-way ANOVA followed by a Dunnett’s post hoc test

Whilst both dabigatran and DAPT with prasugrel effectively inhibited platelet recruitment in healthy ND-fed mice, only prasugrel is part of standard anti-platelet therapy for MI and directly targets platelet activation pathways that are dysregulated in diabetes. We, therefore, chose to further test only DAPT with prasugrel in HFD-fed mice to ensure our findings are directly applicable to the current clinical management of thrombotic risk in PCI patients with diabetes. Body weight increased from 27.6 g ± 2.3 in ND-fed mice to 47.3 g ± 4.9 in HFD-fed mice. Blood glucose levels in HFD-fed mice were 364.38 ± 14.22 mg/dl, a value greater than the 250 mg/dl considered diabetic/hyperglycaemic in mice [12]. LSCI detected a reduction in myocardial perfusion in response to LAD ligation in treated HFD-fed mice as evidenced by the transition to cooler heatmap colours and a concomitant decrease in flux values. However, unlike DAPT-treated mice on a ND, the heatmap colour and flux values recovered to baseline values when the ligature was removed in treated HFD-fed mice indicative of improved perfusion (Fig. 6A–G). Intravital imaging demonstrated less areas devoid of FITC-BSA perfusion in treated HFD-fed mice when compared to treated ND-fed mice suggesting improved FCD in these mice (Fig. 6A).

**Fig. 7:**
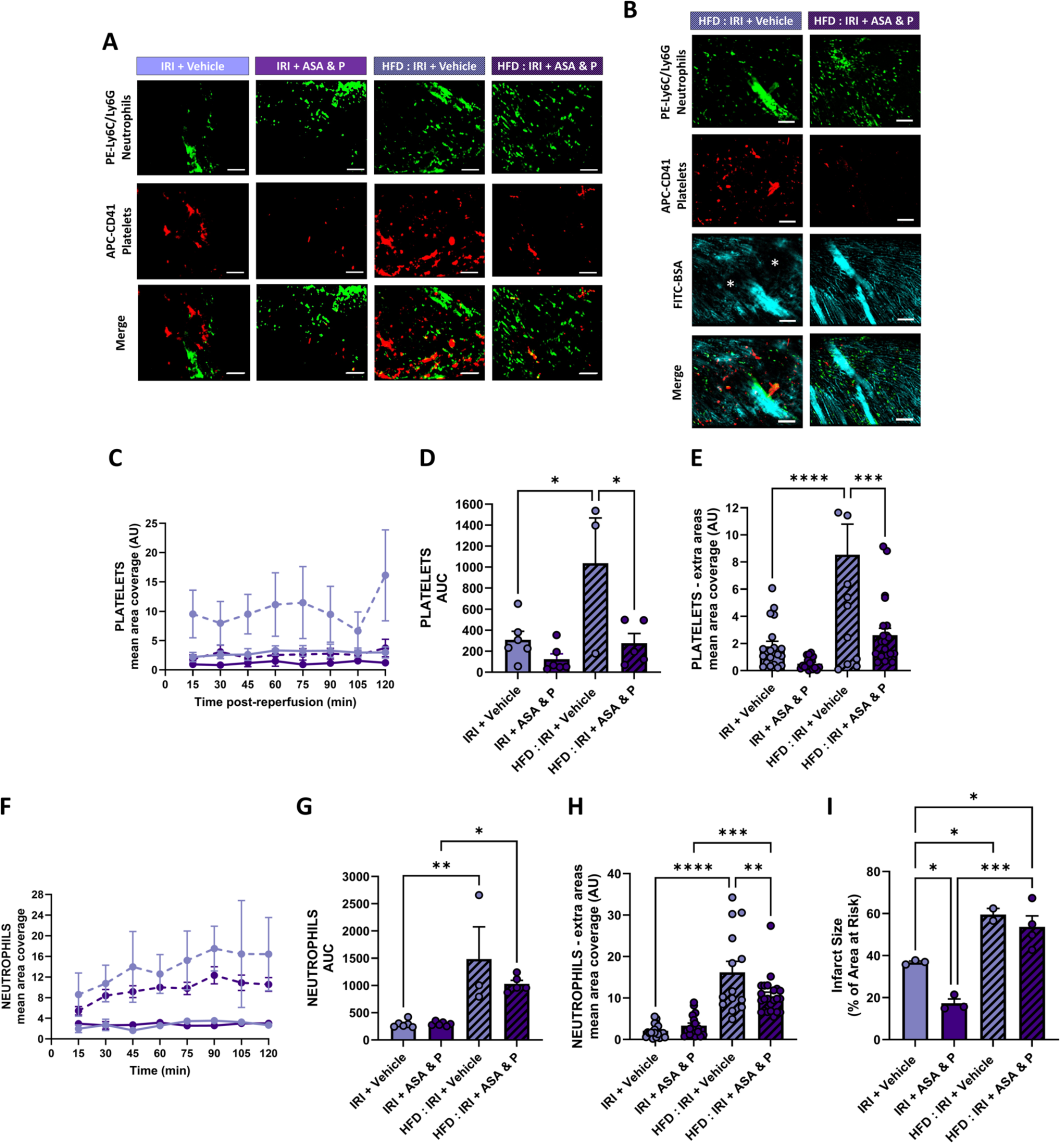
Heightened platelet and neutrophil presence following IRI in HFD-fed mice—DAPT with prasugrel reduces, but does not abolish this, with no effect on infarct size. **A**, **B** Representative intravital images showing neutrophils (green), platelets (red) and FITC-BSA (cyan) at 120 min post-reperfusion. Darker areas devoid of FITC-BSA perfusion are indicated by the asterisk (*). Scale bar represents 10 μm. **C**, **F** Quantitative analysis of intravital data for adherent platelets and neutrophils imaged continuously in a single region over a duration of 120 min post-reperfusion. **D**, **G** Corresponding area under the curves (AUC) for adherent platelets and neutrophils for this continuously imaged area. **E**, **H** Quantitative analysis of intravital data for adherent platelets and neutrophils imaged in extra areas at the end of experimentation. *N* = 2–6/group. **I** Quantitative analysis of infarct size and area at risk (AAR) determined by dual Evan’s blue and TTC staining of heart sections. *N* = 2–4/group. Graphs display mean ± SEM. *p < 0.05, **p < 0.01, ***p < 0.001, ****p < 0.0001 when tested using a one-way ANOVA followed by a Dunnett’s post hoc test

Intravital imaging demonstrated a remarkable increase in the accumulation of platelet aggregates in the injured hearts of HFD-fed mice when compared to mice on a ND. Indeed, relatively larger and occlusive microthrombi were observed surrounded by areas devoid of FITC-BSA perfusion (Fig. 7A, B). DAPT with prasugrel significantly reduced their presence in HFD-fed mice, although their number remained higher than those seen in treated mice fed a ND (Fig. 7A–E). Neutrophil adhesion was also remarkably increased in HFD-fed mice in response to injury to levels significantly higher than injured mice fed a ND. They often formed adherent clusters, making it difficult to identify individual cells. Neutrophil accumulation was also noted in medium-sized blood vessels if these were captured in the imaged field of view and continued to increase over the 2 h imaged reperfusion period. DAPT with prasugrel reduced their presence in HFD-fed mice, although their number also remained higher than those seen in treated mice fed a ND (Fig. 7A, B, F–H).

HFD-fed mice sustained significantly larger infarcts when compared to mice fed a ND (1.62-fold greater). In contrast to the effectiveness of DAPT with prasugrel in mice on a ND, DAPT with prasugrel was ineffective in reducing the infarct size in HFD-fed mice (Fig. 7I).

### DAPT with cangrelor led to excessive bleeding and high mortality

The invasive nature of the open chest surgery to induce IRI and perform beating heart imaging resulted in bleeding in mice treated with anti-platelet therapy but was negligible in non-treated mice. Bleeding was noted at the surgical sites, making subsequent imaging difficult, especially with DAPT with cangrelor. Due to the high rate of bleeding and mortality in IRI mice receiving DAPT with cangrelor (80.0%), doses were reduced from 6 μg/kg/min to 0.5 μg/kg/min. However, excessive bleeding still occurred, resulting in high mortality and an inability to perform intravital imaging. Therefore, no further studies using this DAPT combination were performed.

## Discussion

**Fig. 8:**
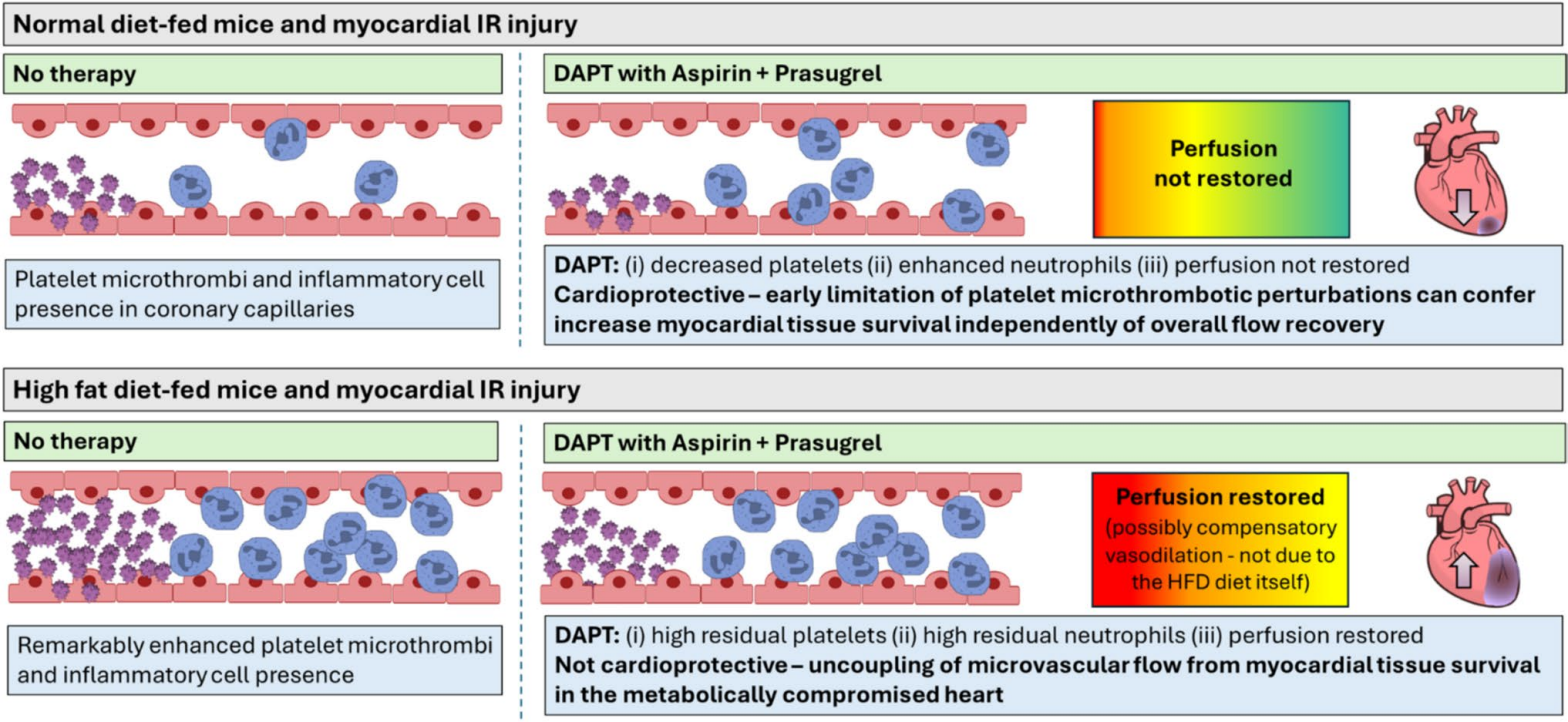
A simple linear relationship between reduced platelet microthrombi, improved perfusion, and infarct limitation is not supported by this study.

Recent evidence suggests the failure to adequately reperfuse the coronary microcirculation, despite successful re-opening of the culprit epicardial artery, may be a stronger predictor of major adverse cardiovascular events than infarct size itself in patients post-MI [43]. Platelet adhesion and aggregation within the smallest blood vessels of the reperfused heart represent one of several pathophysiological mechanisms contributing to persistent coronary microvascular obstruction [27]. Our experimental in vivo study of the beating heart is the first of its kind to provide original mechanistic insights into whether contemporary P2Y_12_ inhibitors can modify thrombotic events specifically taking place at the level of the normal and hyperglycaemic IR injured coronary microcirculation. Intravitally, we demonstrate that the relationship between the microvascular effects of DAPT and infarct size is not always causal. Indeed, our data do not support a simple linear relationship between reductions in microvascular platelet presence, improvements in perfusion, and infarct limitation. In ND-fed mice, reducing platelet aggregates limited the infarct size, despite no improvement in overall ventricular perfusion, and in the presence of increasing inflammatory cell infiltration. These findings suggest that early attenuation of platelet accumulation can confer cardioprotection independently of flow recovery. In contrast, chronic hyperglycaemia profoundly altered this relationship. In DAPT-treated HFD-fed mice, excessive residual platelet presence and heightened neutrophil-driven inflammation dominated the injury response such that infarct size was not reduced despite improved perfusion. This indicated an uncoupling of microvascular flow from myocardial tissue survival in the metabolically compromised heart. Collectively, these findings reveal new mechanistic insights into how metabolic status shapes the effectiveness of anti-platelet strategies in myocardial IRI, highlighting that platelet inhibition is necessary but insufficient to limit infarct size when metabolically driven excessive inflammation is present. These results may help explain the reduced cardioprotective efficacy of DAPTs in patients with T2DM (Fig. 8).

Whilst platelet inhibition immediately post-reperfusion confers cardioprotection independently of flow recovery in otherwise healthy mice, metabolic compromise uncouples microvascular flow from myocardial tissue survival. This may explain the diminished cardioprotective efficacy of DAPTs in patients with T2DM and supports exploring combination vasculoprotective therapies targeting multiple microvascular perturbations.

Although many clinical studies have assessed the ability of P2Y_12_ inhibitors to reduce infarct size, secondary cardiovascular events and mortality, limited studies have compared their effects on microvascular function. This has commonly been assessed using indirect methods such as IMR, a measurement taken from an epicardial artery [47, 48]. IMR reflects how difficult it is for blood to flow through the microvasculature during maximal vasodilation, but cannot reveal the exact biological mechanisms causing any resistance or improvements in flow. Our direct, real-time imaging of the mouse beating heart visualised rapid recruitment of occlusive platelet aggregates, making them amongst the first wave of cells to infiltrate the injured coronary capillaries. Their presence was reduced with DAPTs, with some improvement in FCD when DAPT with prasugrel was used, indicating a key role for capillary microthrombi in impairing overall microvascular reperfusion. Interestingly, our flow cytometry data showed that IRI also ‘primed’ circulating platelets, leaving them in a heightened state of readiness to become fully activated as evidenced by them being more reactive to agonists. The pathological role of such ‘primed’ platelets in mediating cardiac damage was previously demonstrated by the threefold increase in infarct size in mice receiving IRI-activated platelet-rich plasma and the increased cardiac damage in rat hearts receiving platelets from MI patients [67, 71].

A variability in the anti-platelet effectiveness of the tested P2Y_12_ inhibitors was observed in the coronary microcirculation but did not achieve statistical significance. Although DAPT with ticagrelor or clopidogrel showed modest reductions in platelet aggregate deposition, aspirin and prasugrel emerged as the most effective combination, reducing their presence by 2.5-fold (60.0%; continuously observed area) and 3.5-fold (71.1%; extra areas) compared to vehicle treatment. In the clinical assessment of IMR values for clopidogrel, ticagrelor and prasugrel, DAPT with the latter two generally demonstrated a more favourable effect on microvascular function compared to clopidogrel [47, 48, 63]. Indeed, in a rare study comparing all three, Schnorbus and colleagues found that loading with prasugrel improved microvascular function most effectively [54]. Hence, our data demonstrating a somewhat greater efficacy of prasugrel at the microvascular level aligns with these clinical findings. However, it is difficult to make comparisons between our experimental study and clinical studies as the latter did not tend to assess IMR in the immediate aftermath of reperfusion, but usually months after PCI.

Although the platelet P2Y_12_ receptor represents the primary pharmacological target of these agents, the various vasculo- and cardioprotective effects observed in the present study, namely improved myocardial perfusion and reduced infarct size, may extend beyond platelet inhibition alone. Increasing evidence supports platelet-independent therapeutic mechanisms including modulation of cyclooxygenase-2 and prostacyclin (PGI_2_) signalling, which promote vasodilation as well as suppression of the NLRP3 inflammasome [44, 49]. Furthermore, P2Y_12_ inhibitors such as ticagrelor can regulate aquaporin-4 expression and stimulate AMP-activated protein kinase signalling, both of which can attenuate myocardial oedema through increased adenosine formation and subsequent stabilisation of the endothelial barrier [6, 64]. Consistent with this, elevated adenosine levels have been repeatedly demonstrated in both clinical and experimental settings to contribute to the cardioprotective properties of ticagrelor [6]. Collectively, these pleiotropic mechanisms may act synergistically with anti-platelet effects to confer myocardial protection and warrant further investigation.

Targeting an alternative platelet receptor did not offer a superior approach when compared to DAPT with prasugrel. GPIbα, part of the GPIb-IX-V complex, plays a critical role in permitting the initial tethering of platelets to von Willebrand factor immobilised on injured vessels with high shear rates [13]. Therefore, whilst essential for mediating platelet adhesion in arterioles, here we show for the first time that GPIbα could also modify platelet presence in relatively low shear coronary capillaries, reducing platelet presence by 2.9-fold (65.2%; continuously observed area) and 1.4-fold (28.7%; extra areas) compared to vehicle treatment. Although this anti-platelet efficacy was modest, and the effect on ventricular perfusion was similar to DAPT with prasugrel, its pro-inflammatory consequence was greater. This differs from a histological study by Pachel and colleagues in which the same anti-GPIbα Fab reduced neutrophils in the IRI heart 24 h after injury. This was explained by the antibody decreasing the cross-linking of neutrophils to adherent platelets via GPIbα [46]. Intravitally, we noted limited overlap between platelet and neutrophil presence, which may partly explain why concomitant decreases in neutrophil recruitment were not observed.

Neutrophil accumulation generally tended to increase in response to all the antiplatelets tested. Indeed, aspirin alone, aspirin and clopidogrel, and GPIbα inhibition markedly increased neutrophil deposition in IRI coronary capillaries. This worrying increase in neutrophil recruitment is an observation we previously described in the IRI heart using the anti-coagulant heparin [10], and is an effect observed by others in injuries such as peritonitis [3, 70]. We speculate that microthrombi in coronary capillaries may be a double-edged sword [71]. Although detrimental to microvascular flow due to their occlusive nature, their removal may have restored blood flow (mediating a second wave of reperfusion injury) allowing circulating neutrophils access to damaged endothelium primed for their capture. This ssubsequent heightened increase in neutrophil adhesion would have instead reduced perfusion, potentially explaining the LSCI and FCD observations in which blood flow was not restored to baseline values in any anti-platelet-treated mice.

Additional non-classical, yet beneficial, roles for platelets in myocardial IRI have been described. These include the release of stromal cell-derived factor-1α (SDF-1α), transforming growth factor-β1 (TGF-β1) and sphingosine-1-phosphate (S1P), all of which have been implicated in cardioprotective effects [34, 65, 71]. Platelet-derived S1P, in particular, has been shown to limit myocardial IRI by activating the survivor activating factor enhancement (SAFE), reperfusion injury salvage kinase (RISK) and protein kinase B (Akt)/endothelial nitric oxide synthase cardioprotective signalling pathways [34]. Consistent with these findings, Lieder and colleagues demonstrated that platelets could also transmit cardioprotective signals in the context of remote ischaemic conditioning (RIC) [36]. They showed that platelets isolated from volunteers subjected to RIC reduced the infarct size when they were infused into isolated rat hearts undergoing IRI. However, this effect was abolished by aspirin pre-treatment. Collectively, our findings alongside the accumulating evidence for platelet-mediated cardioprotection support a possible dual role for platelets in myocardial IRI. Whilst their accumulation contributes to detrimental coronary microvascular obstruction, platelets may also convey cardioprotective and immunomodulatory signals. Antiplatelets appear to differentially modulate these functions, attenuating microvascular platelet obstruction whilst abrogating platelet-dependent cardioprotection and amplifying neutrophil-driven inflammation. Thus, platelet inhibition may involve a therapeutic trade-off between beneficial and detrimental effects.

It is also possible that the tested therapies were directly pro-inflammatory. Indeed, aspirin can promote neutrophil–endothelial interactions by increasing neutrophil CD11/CD18 adhesion molecules [69]. Alternatively, by preventing the cellular uptake of the potent vasodilator adenosine, P2Y_12_ inhibitors such as ticagrelor may have increased pro-inflammatory cell delivery into coronary microvessels [7, 61]. Ticagrelor can also enhance the pro-inflammatory functions of neutrophils such as chemotaxis and phagocytosis by inhibiting the cellular uptake of adenosine [1]. Regardless of the mechanism, a likely consequence of this accompanying heightened pro-inflammatory response is that the reduced infarct size noted with DAPT with prasugrel may not have been maximal. Indeed, we have previously demonstrated a critical role for inflammatory neutrophils in mediating infarction in this same model [19, 21].

Adhesion of thromboinflammatory cells was not the only detrimental microvascular event. Fibrin was imaged for the first time in vivo in the beating heart, and an abundant capillary presence was observed of varying size and shape. On formation, fibrin polymerises to form a mesh of long, insoluble fibres designed to support platelet aggregation and clot stabilisation. However, limited co-localisation with platelets was observed, with fibrin appearing to be occlusive in its own right as evidenced by no FITC-BSA perfusion in areas downstream of fibrin-rich deposits. Nevertheless, dabigatran reduced not only fibrin, but also platelet presence by 2.9-fold (65.9%; extra areas), indicating that fibrin deposition did contribute to platelet recruitment. Dabigatran has dual effects as it inhibits thrombin-induced platelet activation as well as fibrin formation. Its efficacy, therefore, implies a significant role for thrombin rather than just fibrin formation. Overall, no superior anti-platelet effect, and similar infarct size were observed with dabigatran when compared to DAPT with prasugrel. Persistent capillary presence of neutrophils in dabigatran-treated mice, albeit not increased, was also noted. Whilst others have also shown that dabigatran can limit infarct size, it lacks consistency across species and may have limited clinical translatability due to adverse effects such as increased risk of MI and bleeding [17, 56]. However, alternative approaches to limit thrombin generation such as factor Xa inhibition or contact pathway inhibitors may offer a promising alternative. Overall, our novel data underscore the complexity of coronary microvascular perturbations and highlights the need for therapeutic approaches post-PCI that target not only thromboinflammatory cells, but also components of the coagulation cascade. This may be necessary to increase the salvage of even more viable myocardium.

Elevated blood glucose levels promote a pro-thrombotic state in several ways that include, but are not limited to, endothelial dysfunction, impaired vasotonal responses, leaky capillaries and increased platelet responsiveness to agonists. This results in a greater thrombotic burden in patients with T2DM following an MI. Studies have shown that these patients can have insufficient responses or are resistant to P2Y_12_ inhibitors, which leads to a greater likelihood of stent thrombosis and more extensive microvascular dysfunction post-PCI [2, 25, 28]. Whether the latter is due to a reduced anti-platelet efficacy at the level of the chronically hyperglycaemic reperfused coronary microcirculation is not known. We, therefore, treated HFD-fed mice with DAPT with prasugrel. We first demonstrated a striking heightened presence of platelet aggregates and neutrophils within hyperglycaemic injured coronary capillaries. Microthrombi were larger than those observed in ND-fed mice, occupied longer lengths of the capillaries and markedly reduced FITC-BSA perfusion. Regardless, DAPT with prasugrel was highly effective in reducing platelet presence. Indeed, reductions of 3.8-fold (73.4%; continuously observed area) and 3.3-fold (69.4%; extra areas) were observed when compared to HFD-fed mice receiving vehicle treatment. This data indicated a greater absolute benefit of DAPT with prasugrel in hyperglycaemic mice when compared to its efficacy in ND-fed mice, likely attributed to the increased platelet reactivity and greater pro-thrombotic state in the setting of diabetes. This phenomenon mirrors the results of clinical trials such as TRITON TIMI 38 which also showed aspirin and prasugrel to be especially efficacious in patients with diabetes [66]. Indeed, whilst primary endpoints (cardiovascular death, MI, stroke) were reduced in both non-diabetic and diabetic patients, greater relative reductions were seen in the latter.

Despite this greater anti-platelet efficacy in injured HFD-fed mice, intravitally we showed that platelet and neutrophil presence remained at a higher level than that observed in treated ND-fed mice. Larger infarcts were also noted in hyperglycaemic mice which were not reduced by DAPT with prasugrel. This was despite the unexpected restoration of ventricular perfusion to baseline values which may have been due to a compensatory vasodilation frequently described in early diabetes [29, 59]. Indeed, we have previously demonstrated that mice fed the same HFD for 16 weeks had better coronary vasodilatory responses to acetylcholine compared to ND-fed mice [18]. We speculate that the significant residual thromboinflammatory landscape after DAPT with prasugrel likely led to greater cardiac tissue damage, offsetting the protective effects of improved perfusion under conditions of metabolic stress. This data again highlights the need for both thrombotic and inflammatory cells to be targeted post-reperfusion and particularly so in a chronically hyperglycaemic environment.

## Limitations of the study

Administration of P2Y_12_ inhibitors prior to the induction of injury was necessary to ensure systemic presence of the active drug component during the period of reperfusion injury. It would not have been safe to orally deliver these agents once the mouse was anaesthetised. Whilst this was not ideal, the study did allow us to provide proof-of-concept data that, when available, all tested DAPTs could varyingly and modestly inhibit platelet recruitment. Importantly, it allowed us to highlight their detrimental effects on inflammatory cell presence. Furthermore, it is a particularly relevant model for patients with ischaemic heart conditions who are already on some form of DAPT.

Coronary blood flow is not only influenced by occlusive cellular components and coagulation proteins, but also by myogenic forces impacting vasotone, extravascular compression, oedema and neurohumoral agents. Therefore, an important limitation of this study is that it did not investigate the extent to which each of these, if any, could also have contributed to the observed responses in the normoglycaemic and chronically hyperglycaemia treated and untreated injured microvessels.

Murine microvessels share key features with the human coronary microcirculation, including similar arteriolar–capillary–venular organisation, conserved flow-regulatory mechanisms and common endothelial and smooth muscle signalling pathways (e.g. NO, endothelin, PGI_2_, ROS, shear-dependent responses). Hence, dynamic processes such as leukocyte trafficking, RBC flow patterns and acute responses to ischaemia or inflammation closely resemble those in humans, supporting mouse heart imaging as a relevant model of coronary microvascular physiology. However, translation is limited by important differences: murine microvessels are smaller, show less branching heterogeneity, lack collateralization, function at higher heart rates, and experience minimal compressive forces compared with the largely intramural human circulation. Also, imaging was restricted to superficial murine coronary microvessels in the epicardial and outer myocardial layers. Future studies could incorporate thioflavin-S staining to assess perfusion in deeper myocardial layers [8, 24]. Thus, whilst intravital coronary imaging yields valuable mechanistic insight, these anatomical and mechanical differences should be considered when extrapolating findings to humans.

Finally, although the HFD-fed mouse model captures key aspects of human cardiovascular risk, including obesity, insulin resistance, endothelial dysfunction, inflammation and dyslipidaemia, it does not fully reflect human disease complexity. Mice differ in lipid metabolism, do not develop atherosclerosis without genetic modification and lack many contributing comorbidity factors present in patients, such as ageing, hypertension and lifestyle influences. Hence, this model provides useful insight into the early mechanistic links between metabolic stress and cardiac/coronary microvascular dysfunction, but only partially recapitulates clinical cardiovascular risk.

## Concluding remarks

Most clinical studies focus on the macrovascular outcomes of DAPT such as preventing stent thrombosis and recurrent STEMI. However, our novel research demonstrates that at the level of the coronary microcirculation, where microthrombus burden post-reperfusion is high, DAPT alone is not optimal. We show that DAPTs, whilst effective in reducing infarct size in normoglycaemic settings, may not be maximally salvaging viable myocardium due to the associated increase in inflammatory cell recruitment, presence of occlusive fibrin deposits in coronary capillaries and sub-optimal restoration of ventricular perfusion. In the diabetic setting, further testing of DAPT with prasugrel reveals a significant residual platelet presence, and a much heightened neutrophil infiltration, which likely contributes to the lack of a beneficial impact on the much larger infarcts despite compensatory improvement in perfusion. We, therefore, propose adopting a multi-targeted vasculoprotective approach, or combination therapy, to maximally mitigate cardiac damage, particularly in the presence of a comorbidity. Clinically, this is critical as every 5% increase in infarct size is associated with an approximate 6–7% decrease in ventricular ejection fraction which subsequently correlates with a poorer prognosis [9, 50]. Hence, seemingly small improvements in infarct size can profoundly impact clinical outcomes.

## Supplementary Information

Below is the link to the electronic supplementary material.

Supplementary Figure 1. Absolute values for flux, obtained by LSCI, do not show baseline differences between vehicle treated ND and HFD-fed mice undergoing IRI. LSCI measures relative perfusion rather than absolute flow, as flux is affected by illumination intensity, camera exposure, sensor-to-tissue distance and angle, tissue optical properties, noise, and speckle processing. These factors vary across sessions and subjects, limiting the comparability of absolute values. Baseline normalisation mitigates these confounding factors, enabling meaningful within- and between-subject comparisons. For these reasons, we normalised values to baseline in this study. Here we present the (A) quantitative time-course analysis of absolute flux unit readings obtained during the entire IRI period and (B) an associated bar graph showing absolute flux unit readings for the pre-injury baseline timepoint only. These absolute values show no difference in baseline perfusion between vehicle-treated ND and HFD-fed mice undergoing IRI, suggesting the HFD per se did not alter resting perfusion. Interestingly, ND mice receiving DAPT tended to have higher absolute baseline flux values, an effect that was masked in the normalised data. This could suggest increased basal perfusion in this group. However, due to the confounding factors mentioned above, this difference cannot be conclusively attributed to therapy. Importantly, the cardioprotective effects of prasugrel in ND mice (i.e. smaller infarcts) cannot be inferred solely from absolute flux values and so we have avoided speculation. N=5-9/group. Graphs display mean ± SEM. *p<0.05 when tested using a one-way ANOVA followed by a Dunnett’s post hoc test. Supplementary file1 (TIF 532 KB)

## Data Availability

The authors confirm that the data supporting the findings of this study are available within the article and its supplementary materials. Raw data supporting the findings of this study are available from the corresponding author, Dr Neena Kalia, on request.
