# Supplementary figures and images for: Comparing anti-platelet and anti-thrombin therapies in the ischaemia–reperfusion injured coronary microcirculation of healthy and diabetic mice
Source: Basic Res Cardiol. 2026 Apr 2;121(3):351–70. doi: 10.1007/s00395-026-01168-7 (PMC13186908; doi:10.1007/s00395-026-01168-7)

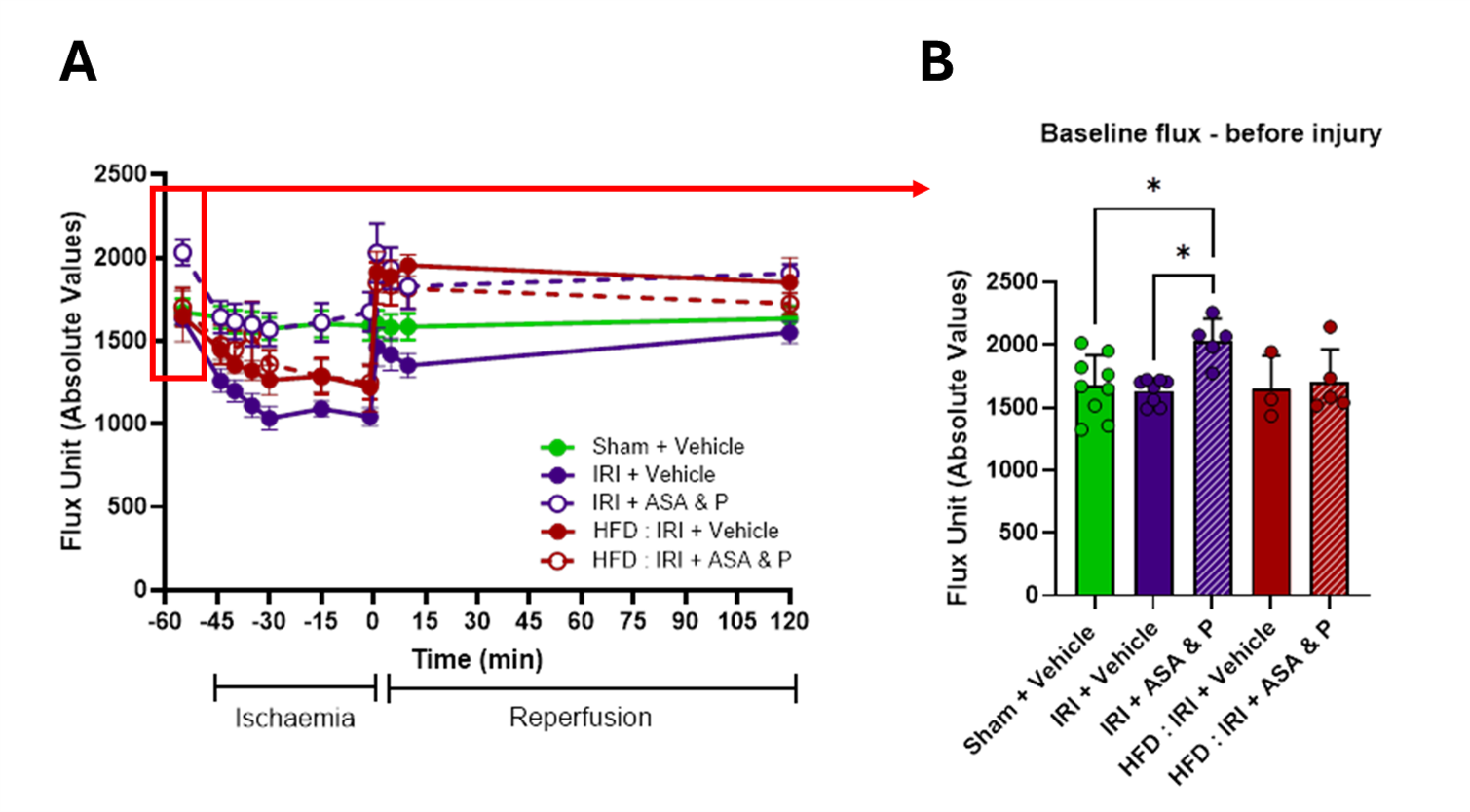

Supplement: Supplementary file 1 — Supplementary Figure 1. Absolute values for flux, obtained by LSCI, do not show baseline differences between vehicle treated ND and HFD-fed mice undergoing IRI. LSCI measures relative perfusion rather than absolute flow, as flux is affected by illumination intensity, camera exposure, sensor-to-tissue distance and angle, tissue optical properties, noise, and speckle processing. These factors vary across sessions and subjects, limiting the comparability of absolute values. Baseline normalisation mitigates these confounding factors, enabling meaningful within- and between-subject comparisons. For these reasons, we normalised values to baseline in this study. Here we present the (A) quantitative time-course analysis of absolute flux unit readings obtained during the entire IRI period and (B) an associated bar graph showing absolute flux unit readings for the pre-injury baseline timepoint only. These absolute values show no difference in baseline perfusion between vehicle-treated ND and HFD-fed mice undergoing IRI, suggesting the HFD per se did not alter resting perfusion. Interestingly, ND mice receiving DAPT tended to have higher absolute baseline flux values, an effect that was masked in the normalised data. This could suggest increased basal perfusion in this group. However, due to the confounding factors mentioned above, this difference cannot be conclusively attributed to therapy. Importantly, the cardioprotective effects of prasugrel in ND mice (i.e. smaller infarcts) cannot be inferred solely from absolute flux values and so we have avoided speculation. N=5-9/group. Graphs display mean ± SEM. *p<0.05 when tested using a one-way ANOVA followed by a Dunnett’s post hoc test. Supplementary file1 (TIF 532 KB) [file 395_2026_1168_MOESM1_ESM.tif]
